# Parameter estimation without confidence intervals?

**DOI:** 10.4178/epih.e2016036

**Published:** 2016-08-18

**Authors:** Jong-Myon Bae

**Affiliations:** Department of Preventive Medicine, Jeju National University School of Medicine, Jeju, Korea

Dear Editor,

Fukuhara and Hori’s article [1] reported the possibility of calculating the incidence of spontaneous intracerebral hemorrhage (sICH) using publicly accessible data in Japan. If the method were valid and satisfied statistical assumptions, overcoming the limitation of the source of the data, known as Diagnosis Procedure Combination (DPC) data, this suggestion could be an innovative method. Because it will not need to operate a population-based registry and to conduct a nationwide survey in order to calculate the disease statistics. However, the article raises three major issues.

First, the incidence is calculated from the newly diagnosed cases among the population of those with the potential to have the target disease. Thus calculating the incidence requires the raw data on the incident case and source population with baseline information on age, sex, time of occurrence, etc. However, DPC data comprises ‘summarized statistics’ based on reimbursement data covering 65.1% of general beds. Thus the authors did calculate the parameter only, without its confidence intervals (CIs). Of course, the authors did not estimate the sex-adjusted and age-adjusted incidence with its CIs. It should be noted, however, that the parameter without its CIs is not a statistically estimated value.

Second, although the authors tried to use the ‘mask rate’ with several assumptions in order to overcome these limitations, DPC data are, fundamentally, not a valid source to be used for calculating an incidence. Notably, using contaminating or incomplete incident cases based on the authors’ description is the fatal limitation on estimating an incidence. Using the mask rate alone could not guarantee the statistical inference of an unknown sICH incidence.

Last, the author insisted that ‘prefectural mortality due to sICH and prefectural sICH incidence in the DPC database were both consistent over the years’. However, the correlation or consistency between the calculated incidence and the mortality rate does not guarantee the reliability of the DPC data. On the other hand, Figure 2B showed that the relationship between incidence and crude mortality was not statistically significant (r= 0.22, p=0.14). That means the DPC database has a serious limitation in that it cannot be used to estimate the incidence value.

In summary, the authors described the purpose of this research as examining the adequacy of utilizing publicly accessible DPC data. Based on Figure 2B as well as contaminating or incomplete incident cases based on the authors’ description, the authors should conclude that the DPC data is worthless in estimating the incidence of sICH in Japan.

## References

[1] Fukuhara T, Hori YS (2016). Prefectural difference of spontaneous intracerebral hemorrhage incidence in Japan analyzed with publically accessible diagnosis procedure combination data: possibilities and limitations. Epidemiol Health.

